# A predicted structure of NADPH Oxidase 1 identifies key components of ROS generation and strategies for inhibition

**DOI:** 10.1371/journal.pone.0285206

**Published:** 2023-05-03

**Authors:** Yezhou Liu, Shiyu Liang, Danfeng Shi, Yue Zhang, Chen Bai, Richard D. Ye

**Affiliations:** 1 Kobilka Institute of Innovative Drug Discovery, School of Medicine, The Chinese University of Hong Kong, Shenzhen, Guangdong, China; 2 Shenzhen Bay Laboratory, Shenzhen, Guangdong, China; 3 Warshel Institute of Computational Biology, School of Medicine, The Chinese University of Hong Kong, Shenzhen, Guangdong, China; 4 The Chinese University of Hong Kong, Shenzhen Futian Biomedical Innovation R&D Center, Shenzhen, China; University of Botswana, BOTSWANA

## Abstract

NADPH oxidase 1 (NOX1) is primarily expressed in epithelial cells and responsible for local generation of reactive oxygen species (ROS). By specifically manipulating the local redox microenvironment, NOX1 actively engages in epithelial immunity, especially in colorectal and pulmonary epithelia. To unravel the structural basis of NOX1 engaged epithelial immune processes, a predicted structure model was established using RaptorX deep learning models. The predicted structure model illustrates a 6-transmembrane domain structure, a FAD binding domain, and an NADPH binding/NOXO1 interacting region. The substrate/cofactor binding scheme with respect to this proposed model highly correlates with published reports and is verified in our site-directed mutagenesis assays. An electron transport chain, from NADPH to FAD and the two heme groups, was well supported by the predicted model. Through molecular docking analysis of various small molecule NOX1 inhibitors and subsequent experimental validation, we identified pronounced active sites for potent NOX1 inhibition. Specifically, LEU60, VAL71, MET181, LEU185, HIS208, PHE211, TYR214, and TYR280 in the transmembrane domain form an active pocket for insertion of the small molecule inhibitors to inhibit electron transfer between the heme groups, thus affecting extracellular ROS generation. Altogether, our study provides structural information to help elucidate the role of NOX1 in epithelial generation of ROS and sheds light on the development of therapeutics for NOX1 related illnesses.

## Introduction

NADPH oxidases (NOX) are a family of membrane-bound enzymes with the specific function of producing reactive oxygen species (ROS) via electron transfer from nicotinamide adenine dinucleotide phosphate (NADPH) [1,2]. Reactive oxygen species (ROS) are oxygen-derived molecules, which form an oxidation cascade, and actively contribute to oxidative stress, cell damage, clearance of bacterial pathogens as well as other physiological and pathological processes. So far, seven NOX isoforms have been discovered, including NOX1-5, DUOX1 and DUOX2. Besides, soluble subunits of the NOX complexes have been identified, including the organizer subunits p47^phox^ and NOXO1, the activator subunits p67^phox^ and NOXA1, and DUOX-specific maturation factors DUOXA1 and DUOXA2 [1,2].

Among all NOX isoforms, NADPH oxidase 1 (NOX1) and neutrophil NADPH oxidase (NOX2) show the highest homology of their protein sequences, sharing around 60% of similarity [2]. The activation of NOX1 and NOX2 both requires the organizer subunits p47^phox^ for NOX2 and its structural homolog, NOXO1 for NOX1, the activator subunits p67^phox^ for NOX2 and its structural homolog, NOXA1 for NOX1, as well as other factors including p22^phox^ and the Rac small GTPase [3]. Homologous analysis revealed that, for the organizers p47^phox^ and NOXO1, the latter lacks an autoinhibitory region (AIR), explaining the constitutive activation of NOX1 complex [1,4]. Studies in cardiovascular cells also indicated that p47^phox^ could substitute the role of NOXO1 to activate NOX1, and this may be attributed to structure similarities between these homologs [5,6]. Specifically, during NOX1 activation, NOXO1 provides a scaffold linking NOXA1 and p22^phox^, which enables the interaction between NOXA1 and NOX1, a prerequisite for the activation of NOX1. Binding of the NOX1 co-factors, such as heme and flavin adenine dinucleotide (FAD), as well as active substrates such as NADPH are essential for electron transport and ROS generation [1,7,8].

While NOX2 (also known as gp91^phox^) plays an active role in phagocytic immunity, NOX1 is primarily expressed in epithelial cells and engages in epithelial immunity [2,9–13]. Meta-analysis using public clinical database suggests that NOX1 expression is enriched in the pathogenesis of cancers of epithelial tissues, vasculitis and pneumonia, whereas NOX2 deficiency leads to chronic granulomatous disease (CGD), a primary immunodeficiency leading to defective clearance of invading bacteria [2,12,14–16]. Extensive *in vivo* and clinical discoveries have been made, indicating that NOX1 is crucial in intestinal immunity, especially in IBD and colon cancers. NOX1 promotes mucosal repair in gastrointestinal tract through a novel epithelial FPR1-dependent redox pathway, and also promotes colon epithelial cell migration [17–19]. In addition, NOX1 has also been shown to affect the pathogenesis of various cardiovascular diseases [20]. Since infection and inflammation of respiratory tract, gastrointestinal tract and cardiovascular epithelial and endothelial tissues impose great threats to human health, there has been a great effort in the research of NOX1 as a valuable drug target. As a result, several potent NOX1 inhibitors such as ML171 and GKT137831 have been discovered and are currently in clinical trials [18,21,22].

Despite growing interest in NOX biology, very limited information about the structure of the NADPH oxidases has been available until recently. A crystal structure of NOX5 complex in *Cylindrospermum stagnale* was first resolved in 2017 [23]. A recently published article identified the cryo-EM structure of mouse DUOX1-DUOA1 complex [24]. Another promising structural study of human DUOX1 complex was also published recently [25]. More recently, the cryo-EM structure of the core human NOX2 has been reported [26,27]. The resolved structure demonstrates a topology of six-transmembrane region of NOX2 and a four-transmembrane fold of p22^phox^ subunit. However, the active complex structures of NOX1 and NOX2, comprising of multiple transmembrane and cytosolic subunits, have not yet been resolved. Owing to a rapid development in computational biology, *in silico* protein structure prediction utilizing deep learning approaches shows an increasing accuracy and fidelity. In this study, we developed a RaptorX deep learning-predicted *in silico* structure model of NOX1, cross-validated the RaptorX-predicted model with other deep learning-predicted NOX1 models and experimentally resolved atomic models of other NOX isoforms, and further validated the proposed structure using molecular docking and site-directed mutagenesis. Using the newly built model, we examined the interaction of several potent NOX1 inhibitors by molecular docking in an effort to enlighten their inhibitory mechanisms. Our results show a high degree of correlation between the results from our molecular docking and available data from biochemical studies, implying a useful model predicted by deep-learning methods.

## Materials and methods

### Reagents

Restriction enzymes for molecular cloning were purchased from New England BioLab (New England Biolabs, Ipswich, MA, USA). KOD-Plus-Neo PCR kit was purchased from Toyobo (Osaka, Japan). ClonExpress Ultra One Step Cloning Kit was purchased from Vazyme Biotech (Vazyme Biotech, Nanjing, China; C115). Plasmid mini prep kit was purchased from Tiangen (TIANGEN, Beijing, China; DP103). For cell cultures, DMEM medium was purchased from Gibco (Thermo Fisher Scientific, Waltham, MA, USA), FBS was purchased from Hyclone (GE Healthcare Life Sciences, Chicago, IL, USA), Lipofectamine 3000 transfection reagent kit was purchased from Invitrogen (Invitrogen, Carlsbad, CA, USA). For ROS detection assays, HBSS was purchased from Gibco (Thermo Fisher Scientific), BSA was purchased from Mpbio (MP Biomedicals, Irvine, CA, USA), isoluminol, HRP and PMA were purchased from Sigma-Aldrich (St. Louis, MO, USA). For NOX1 inhibition, ML171 was purchased from Selleck Chemicals LLC (Houston, TX, USA; S5304), VAS2870 was purchased from Sigma-Aldrich (St. Louis, MO, USA; SML0273), GKT137831 was purchased from Selleck Chemicals LLC (Houston, TX, USA; S7171).

### Plasmid constructs

Plasmids with cDNA of human *NOX1*, *NOXO1* and *NOXA1* on pcDNA3.1+ vector were kindly gifted from Prof. J. D. Lambeth at Emory University School of Medicine. Subsequential processing for plasmid amplification was conducted and then subjected to non-endotoxin plasmid mini prep procedure with plasmid mini prep kit following the manufacturers’ instructions. Molecular cloning for generating NOX1 mutants was conducted using ClonExpress Ultra One Step Cloning Kit (Vazyme Biotech), following the manufacturer’s instructions.

### Cell cultures and transfection

HEK293 cells were purchased from ATCC (Manassas, VA, USA; CRL-1573). Cells were seeded 24 hrs before transfection at 2 × 10^5^ cells/well. Transient transfection was conducted using the previously described wild-type/mutant plasmids in a 12-well plate using Lipofectamine 3000 transfection reagent (Invitrogen) following the manufacturer’s instructions. Three plasmids were used for co-transfection: Wild-type and mutant NOX1 in pcDNA3.1-NOX1 (500 ng/well of cells), pcDNA3.1-NOXA1 (270 ng/well) and pcDNA3.1-NOXO1 (230 ng/well). Cells were harvested 24 hrs after transfection for subsequent procedures.

### Western blotting

To check for expression of the transfected NOXA1 and NOXO1, transfected cells were collected and lysed using RIPA lysis and extraction buffer (Beyotime Biotechnology, Nantong, China; #P0013C). Protein concentration was determined by a Thermo Scientific™ NanoDrop™ One Spectrophotometer (Thermo Fisher Scientific; 13-400-518) using Protein A280 method. After SDS-PAGE, the protein samples (10 μg) were transferred to PVDF membranes (Millipore, Billerica, MA, USA; IPVH00010) following manufacturer’s instruction for immunoblotting. Tris-buffered saline containing Tween-20 (TBST) was made with a final concentration of 20 mM Tris, 150 mM NaCl and 0.1% Tween-20 (Sangon Biotech Co., Ltd., Shanghai, China). After an hour’s blocking with 5% (w/v) non-fat milk in TBST, membranes were incubated with primary antibodies at 4°C overnight: Rabbit anti-human NOXA1 IgG polyclonal antibody (Boster Biological Technology, Wuhan, China; #BA2822-2; 1:1000 dilution), mouse anti-human NOXO1 IgG monoclonal antibody (Santa Cruz Biotechnology, Santa Cruz, CA, USA; sc-390927; 1:1000 dilution), β-Actin (8H10D10) Mouse mAb (Cell Signaling Technology, Inc., Danvers, MA, USA; #3700; 1:2500 dilution). After primary antibody incubation, membranes were washed three times with TBST. Membranes were then incubated with the following secondary antibodies at room temperature for 1 hr: Anti-rabbit IgG, HRP-linked Antibody (Cell Signaling Technology, Inc.; #7074; 1:5000 dilution), Anti-mouse IgG, HRP-linked Antibody (Cell Signaling Technology, Inc.; #7076; 1:5000 dilution). Membranes were then visualized using chemiluminescent substrate ECL Western Blotting Detection Reagent (GE Healthcare; RPN2106). Images of blots were captured by a Amersham Imager 600 (GE Healthcare). Quantification of protein bands was performed by densitometry using Image J software (Wayne Rasband, National Institutes of Health).

### *In silico* model construction

Owing to difficulties in stable protein expression, we established *in silico* models of human NOX1. Model was created by RaptorX, an online server for protein structure prediction using sequential information [28]. The algorithm was based on deep learning algorithm, using the prediction of local contact maps or distances between amino acid residues to search for optimal conformations (detailed computational algorithms can be found in [28]). The predicted model was then refined using UCSF Chimera [29]. We also generated a tFold predicted structural model using a deep learning contact prediction algorithm in parallel to the RaptorX structural model [30]. Given that the source code of AlphaFold has been recently released, we further compared our RaptorX structure model with an AlphaFold-predicted model [31]. The RaptorX model along with the other two predicted models of NOX1 showed high confidence, well presented the 6 transmembrane loops, intracellular FAD- and NADPH- binding domains of NOX1. The structures were visualized by PyMOL (Version 2.0, Schrödinger, LLC, New York, NY, 2021). The electron transport tunnel was visualized by CAVER 3.0.3 PyMOL plugin [32].

### Molecular docking

The structural model of NOX1 was prepared using the AutoDock Tool [33]. Hydrogen atoms were added to NOX1 model prior to docking runs. The docking grid of NOX1 was centered on the centroid of the protein model, encompassing the whole protein model coordinates. 3D structural information of NOX1 inhibitors of interest were obtained from PubChem database for docking analysis [34]. AutoDock Vina was employed for the molecular docking procedure [35]. The basis of molecular docking consists of the searching algorithm that generates different ligand binding conformations, and the scoring function that evaluates binding poses based on a force-field [36]. For every small molecule inhibitor, the top-scored conformation generated by docking was selected. The final binding pose was determined with respect to the selected conformation. Interacting sites were searched using the AutoDock Tool. Binding results were presented using PyMOL.

### Free energy calculation

The MM-GBSA module in the Schrödinger software (Maestro, Schrödinger, LLC, New York, NY, 2021) was applied to evaluate the free energy of binding. For the obtained protein-small molecule drug complex, we checked the correctness of the small molecule structure, and performed protonation treatment on the protein complex system under pH 7.0, and deleted all the atomic clashes through structural optimization. In this way, the VSGB solvent model was used to calculate the free energy of binding between the protein and the small molecule through structure minimization sampling [37].

### Mutagenesis analysis

From TCGA database and published clinical case reports of IBD, we identified several amino acid substitution sites in *NOX1* gene from cohorts of colon cancer patients and IBD patients [38]. We also chose several mutations reported in *NOX2* gene of CGD patients for cross-validation [39]. After comparing with our docking results, mutation sites for mutagenesis analysis were determined (Table 1). pcDNA3.1(+)-NOX1 was used as the template for molecular cloning manipulation. The mutations were introduced using overlap extension PCR with elaborately designed primers containing the mutation sites (GENEWIZ, Nanjing, China). Two fragments of NOX1 separated at mutated positions were assembled into restriction enzyme linearized pcDNA3.1(+) vectors with the ClonExpress Ultra One Step Cloning Kit (Vazyme Biotech, C115). Plasmids with NOX1 mutations were further confirmed by DNA sequencing (GENEWIZ). Cell surface expression of NOX1 mutants was analyzed by flow cytometry on an Accuri C6 Plus flow cytometer (Becton Dickinson, Franklin Lakes, NJ, USA), using a mouse anti-human NOX1-FITC antibody (Santa Cruz Biotechnology, Santa Cruz, CA, USA, sc-518023; 1:50 dilution with PBS). Mean fluorescence intensity was analyzed for cell surface expression of the NOX1 mutants.

**Table 1:** Selected sites for mutagenesis analysis.

Mutation Sites	Predicted Functions	Clinical Implication
**P.54 R->M**	HEME Binding	CGD patient NOX2 mutation.
**P.57 A->E**	HEME Binding	CGD patient NOX2 mutation.
**P.60 L -> A**	Inhibitor Binding	N/A
**P.71 V -> A**	Inhibitor Binding	N/A
**P.122 N->H**	HEME Binding	IBD patient NOX1 mutation.
**P.181 M -> A**	Inhibitor Binding	N/A
**P.211 F -> A**	Inhibitor Binding	N/A
**P.214 Y -> A**	Inhibitor Binding	N/A
**P.260 P -> A**	Inhibitor Binding	N/A
**P.280 Y -> A**	Inhibitor Binding	N/A
**P.339 P->H**	FAD Binding	CGD patient NOX2 mutation.
**P.356 R->P**	FAD Binding	CGD patient NOX2 mutation.
**P.360 D->N**	FAD Binding	Common NOX1 polymorphism. Associated with UC in Ashkenazi Jewish males.

### ROS detection assays

Cells were harvested and resuspended with HBSS with Ca^2+^ and Mg^2+^ containing 0.05% BSA. Cells (200 μL) were collected and plated into a 96-well plate (2 × 10^4^ cells/well). To detect the extracellular ROS released by NOX1 on the plasma membrane only, isoluminol was then added into the cells for a final concentration of 50 μM, and HRP as well for a final concentration of 8 U/mL. Cells were incubated at 37°C for 5 mins in a complete light-avoidance environment. The chemiluminescence signals were detected using EnVision 2105 multimode plate reader (PerkinElmer, Waltham, MA, USA). Baseline signals were read for 5 mins. Then PMA (200 ng/mL) substrate and HBSS control were added to the cells, respectively. Stimulated chemiluminescence signals were detected for 1 hr right after the addition of stimulants. For inhibitor assays, cells were plated and pretreated with corresponding drugs at 10 μM 1 hr prior to ROS detection assays.

### Data analysis and statistics

Data were analyzed and represented with GraphPad Prism 8.2.1 (La Jolla, CA, USA). For ROS generation assays, ordinary one-way Analysis of Variance (ANOVA) with a Tukey’s multiple comparisons test was performed among groups of mutants and wild-type. Analysis of inhibitor-treated ROS responses used ordinary one-way ANOVA with a Dunnett’s multiple comparisons test to verify the changes of ROS generation with respect to the wild-type control. To examine the WT and mutant NOX1 expression on cell membrane upon treatment of different inhibitors, ordinary two-way ANOVA was introduced with a Dunnett’s multiple comparisons test with respect to the wild-type control. For WT and mutant NOX1 expression on the cell membrane without inhibitor treatment, ordinary one-way ANOVA with a Dunnett’s multiple comparisons test was performed. ROS generation readouts were calibrated against the expression level of the respective WT and mutant NOX1. To analyze the immunoblot quantification results, the expression densitometry readouts were calibrated with respect to β-actin control, and ordinary two-way ANOVA was further performed between expression of NOXA1 and NOXO1 among wild-type and mutants with a Tukey’s multiple comparisons test. P values were calculated and *p < 0.05, **p < 0.01, ***p < 0.001 and ****p < 0.0001 values were considered as statistically significant. Cellular assays were repeated for at least three times. All numerical data were presented as means ± SEM.

## Results

### *In silico* structure of the human NOX1

**Fig 1:**
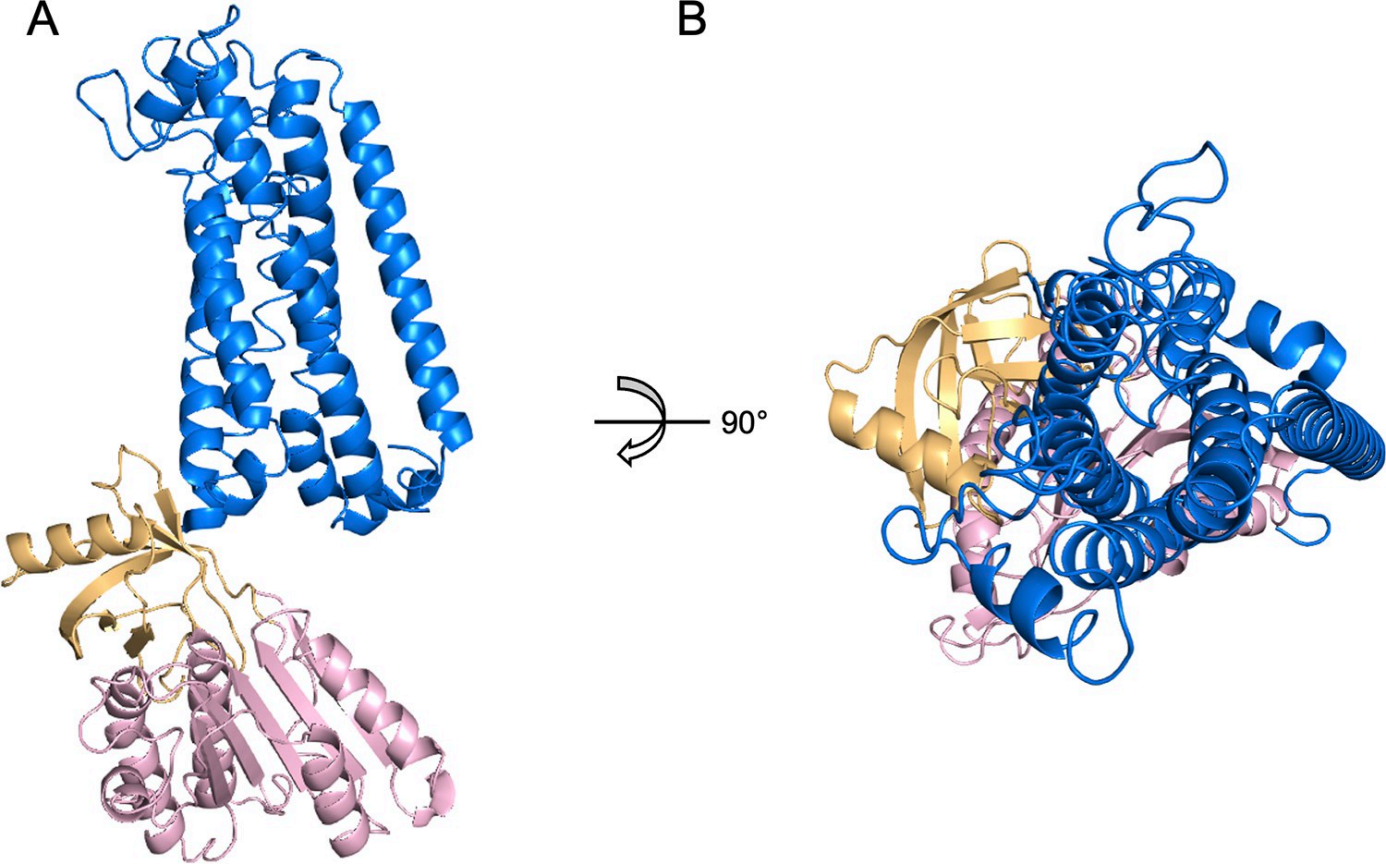
RaptorX-predicted *in silico* structure model of human NOX1. **(A)** Side view of NOX1 *in silico* structure model. **(B)** Extracellular view (bird’s eye view) of NOX1 model. Marine blue represents 6-TM loop domain, golden represents FAD binding domain and light pink for NADPH binding/NOXO1 interacting region.

**Fig 2:**
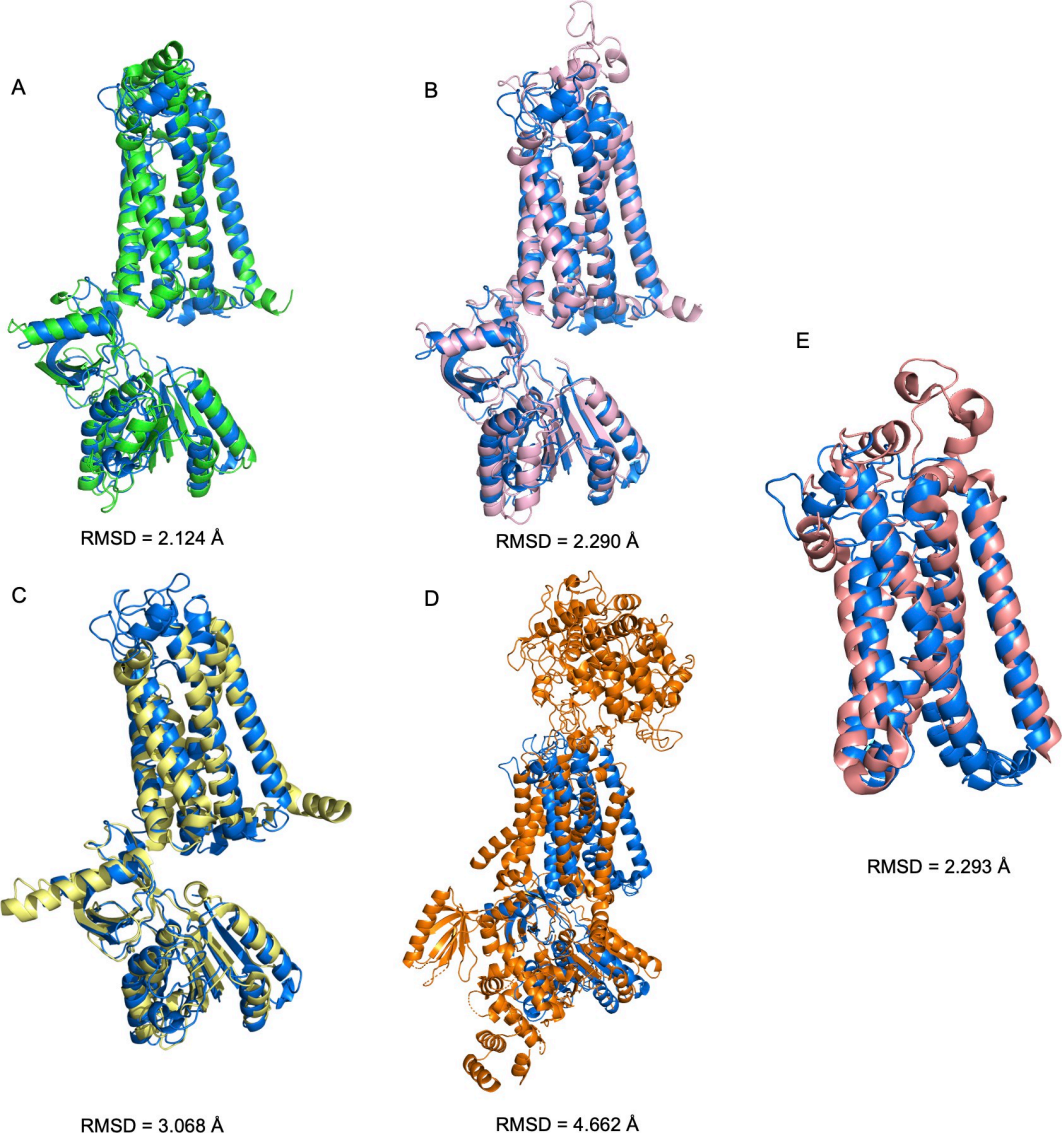
Comparison of predicted models of human NOX1 and experimentally resolved models of csNOX5, human DUOX1 and core human NOX2 transmembrane region. **(A)** Superimposed view of RaptorX-predicted (in dark blue) and tFold-predicted (in green) human NOX1 model. **(B)** Superimposed view of RaptorX-predicted (in dark blue) and AlphaFold-predicted (in pink) human NOX1 model. **(C)** Superimposed view of RaptorX-predicted human NOX1 model (in dark blue) and previously reported csNOX5 model (PDB ID: 5O0T for transmembrane domain, PDB ID: 5O0X for dehydrogenase domain, shown in yellow). **(D)** Superimposed view of RaptorX-predicted human NOX1 model (in dark blue) and previously reported human DUOX1 model (PDB ID: 7D3E, chain A, shown in orange). Root mean square distance (RMSD) were calculated and shown for structural similarity. **(E)** Superimposed view of RaptorX-predicted human NOX1 model (in dark blue) and human NOX2 transmembrane region model (PDB ID: 7U8G, chain A, shown in salmon red).

The NOX1 structure has not been resolved yet. Previous studies, however, have predicted that NOX proteins possess multi-transmembrane domains based on sequence analysis [8]. It was also predicted that both the N-terminus and the C-terminal fragment of NOX1 with the FAD binding domain and the NADPH binding/NOXO1 interacting domain locate intracellularly. This 6-transmembrane domain structure was recently confirmed with the solved atomic models of NOX5 and DUOX1 (PDB ID 5O0T, ID 5O0X, ID 7D3E and ID 7D3F). Using the available sequence data, we constructed an *in-silico* model of human NOX1 protein structure using RaptorX (Fig 1). The NOE violation of the model was 4.0 Å. The predicted local distance map is shown in S1 Fig. We further compared this RaptorX model with another two deep learning-predicted models generated by tFold and AlphaFold, respectively (Fig 2A and 2B). The superimposed illustration demonstrates high structural similarities between those independently predicted models (overall RMSD < 3.0 Å). We also compared the RaptorX model with experimentally resolved atomic models of *Cylindrospermum stagnale* NOX5, human DUOX1 and the core human NOX2 transmembrane region [27] that became available after our model was built initially (Fig 2C–2E). Pronounced shared features were present among NOX isoforms, including the 6-transmembrane domains and the dehydrogenase domains.

### Identification of Heme, FAD, and NADPH binding sites

**Fig 3:**
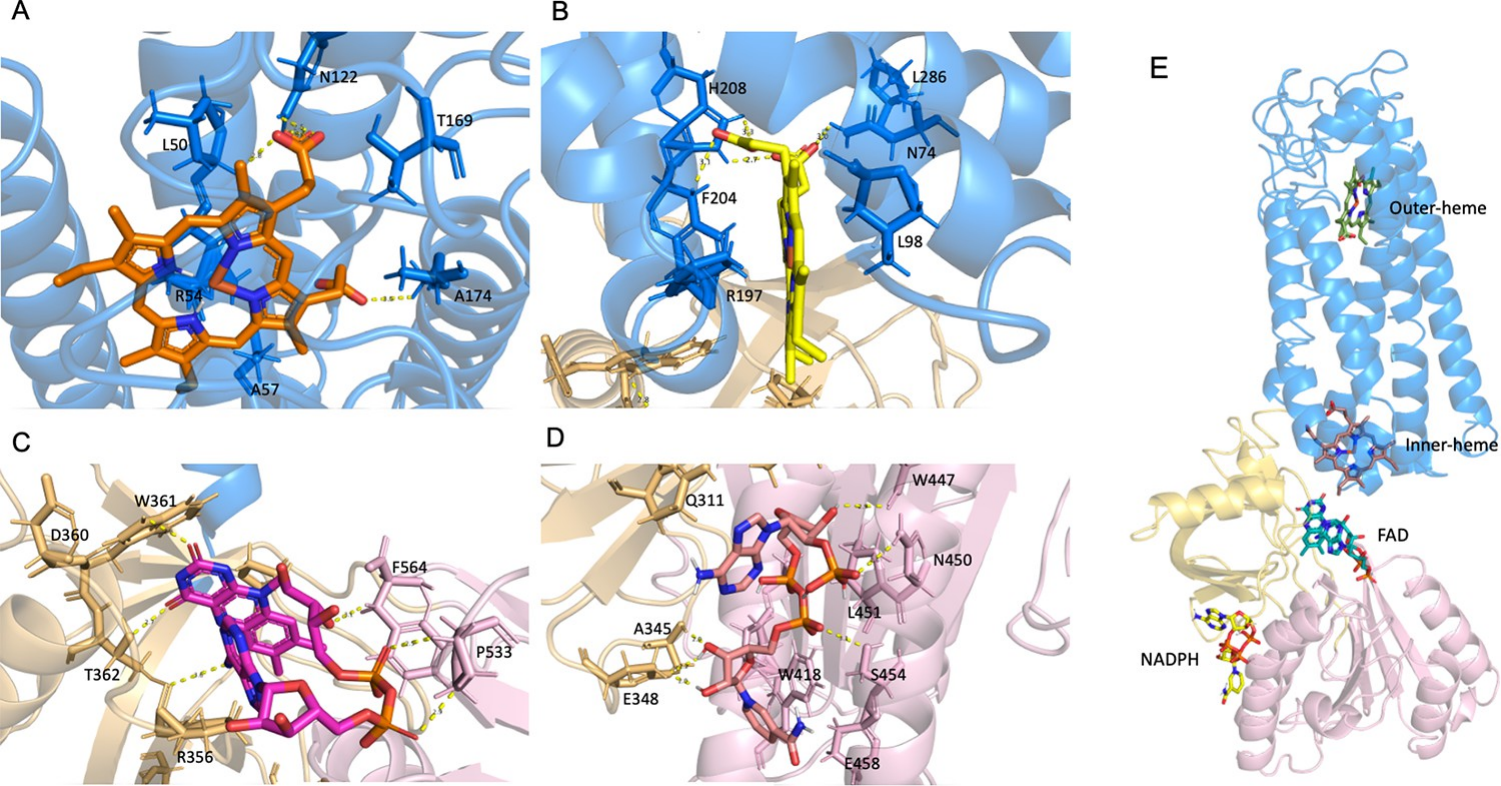
Binding schemes of the heme group, FAD and NADPH to NOX1. **(A) & (B)** Binding poses of heme groups into the 6-TM loop domain of NOX1. **(C)** Binding of FAD into the C-terminus intracellular FAD binding domain of NOX1. **(D)** Binding of NADPH into the C-terminus intracellular NADPH binding domain of NOX1. **(E)** Overview of the heme group, FAD and NADPH binding to NOX1.

Molecular docking procedure was conducted to verify the predicted structure. Since heme and FAD act as crucial co-factors and NADPH as an important substrate for NOX1 activity and ROS generation, we applied heme group, FAD and NADPH as the docking substrates to the RaptorX model (Fig 3). The results indicated possible active sites of the modeled structure with these substrates. Heme group assumes a posture deep into the transmembrane loop domain of NOX1. Specifically, two heme binding pockets were discovered, one closer to the extracellular environment (Fig 3A) and the other next to the intracellular region of the 6-TM loop (Fig 3B). The extracellular-adjacent one (outer-heme) was found to interact with residues ALA57, TRP272, PRO260, ARG241, ASN61, ALA174, TYR33, LEU50, ARG54, ASN122, and THR169. The one closer to the intracellular compartment (inner-heme) resides in the pocket formed by PHE201, HIS101, ARG198, LEU98, ASN74, ARG197, TRP337, and PHE204. Of note, short distance hydrogen bonding was established between the outer-heme and ASN122, ILE173 and LEU50, as well as the inner-heme and ASN74, HIS209 and PHE204. FAD interacts with the C-terminal FAD binding domain of the modeled NOX1 structure (Fig 3C), consisting of residues PHE340, PHE564, ARG356, THR341, ARG440, ALA358, PRO533, ILE405, TRP361, and PRO339. NADPH assumes an intensive interaction with adjacent residues LEU451, TRP418, ASN450, GLN311, ALA345, SER454, GLU458, ALA411, LYS415, THR343, TRP447, and GLU348 (Fig 3D). These results are further corroborated using models predicted by tFold and AlphaFold algorithms (S1 and S2 Tables). The aforementioned results were consistent with published literature of biochemical studies suggesting the involvement of these active residues in binding of heme, FAD and NADPH.

### Electron transport chain of the *in silico* structure

**Fig 4:**
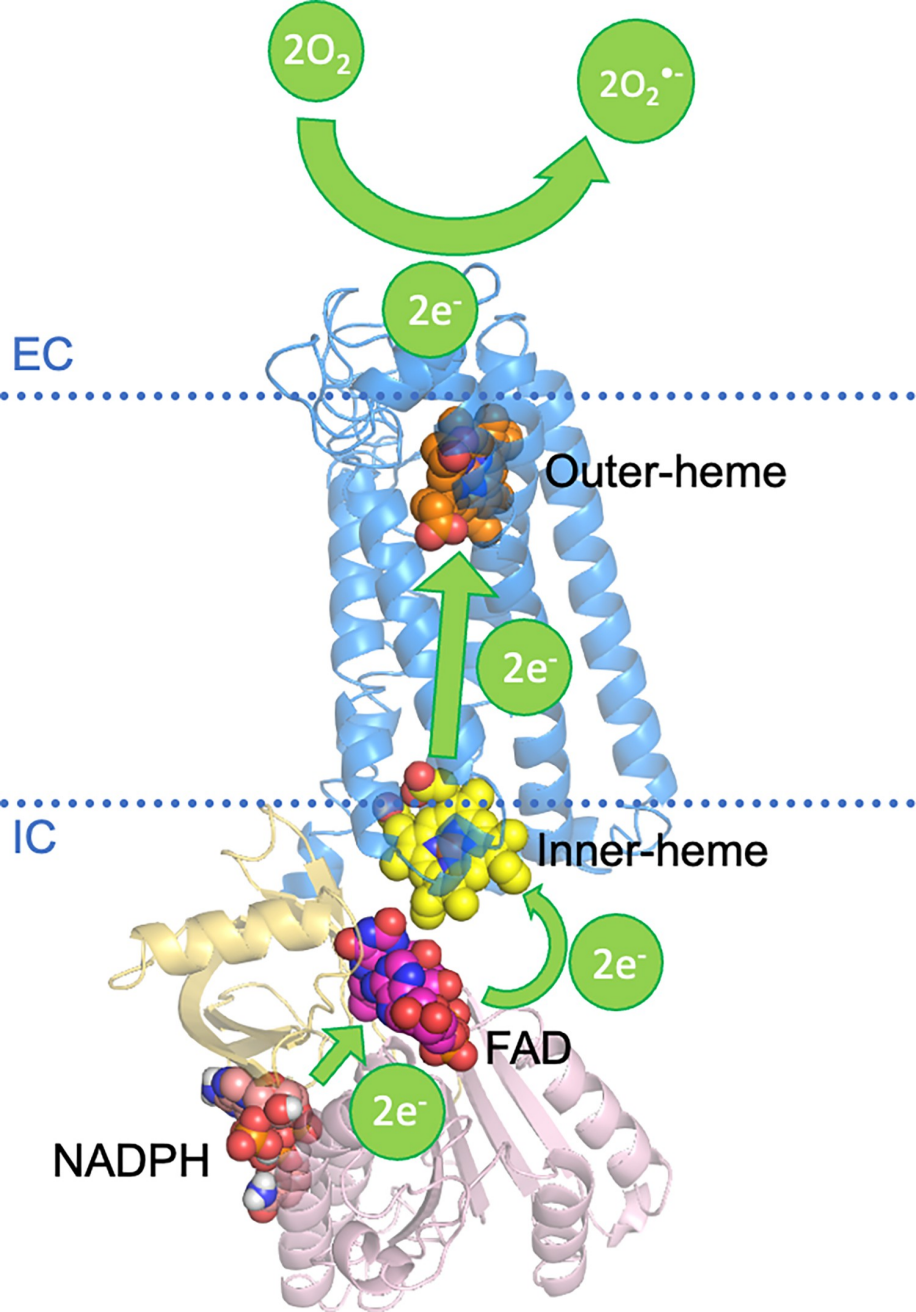
Schematic illustration of electron transfer in NOX1. The electrons are donated by an NADPH at the NADPH interacting domain of NOX1. A FAD molecule then accepts the two electrons released from the NADPH molecule, and is reduced to FADH_2_, which further passes two electrons on to the transmembrane heme groups. The electrons are then transported through the 6-TM loop region towards the extracellular part of NOX1. The extracellular region provides a harbor for the final step of ROS generation, where two oxygen molecules encounter two electrons released by the outer-heme group and are reduced into the free radical form of O_2_^•−^.

After deciphering the active binding sites of important enzyme substrates involved in electron transport of NOX1 and ROS production with our *in silico* structure, we now have a foundation of the crucial electron transport chain of NOX1. The overall reaction of ROS generation can be simplified as the transfer of 2 electrons from an intracellular NADPH molecule to two extracellular oxygen molecules [40], as is illustrated in Fig 4.

The active electron and proton transport scheme described above is consistent with published literature. Previous studies have shown that histidine residues HIS101, HIS115, HIS209 and HIS221 were described as crucial residues supporting the proton transport in the 6-TM loop of NOX1 [7]. In our predicted model, these histidine residues point towards the center of the 6-TM loop, providing necessary space for proton transport. A model of transmembrane proton and electron tunnel is hence demonstrated in S2 Fig.

### Mutational assays of NOX1 verified the *in silico* structure

Several NOX1 mutations have been identified based on publicized data [17,38]. We compared the mutations of the *NOX1* genes from IBD and colon adenocarcinoma patients with the key binding residues identified in our docking analysis using the NOX1 structural model [12,17,38,41]. Strikingly, our docking results overlap with these reported mutations in patients with IBD and colon adenocarcinoma. To identify additional mutation sites, we further searched for mutations of *NOX2* gene in CGD patients. Owing to their sequence similarity, we believe that they might be similar in their structures and key functional sites. Interestingly, some NOX2 mutations reported in CGD patients are remarkably similar to our NOX1 docking results, suggesting potentially novel NOX1 mutations in IBD patients.

**Fig 5:**
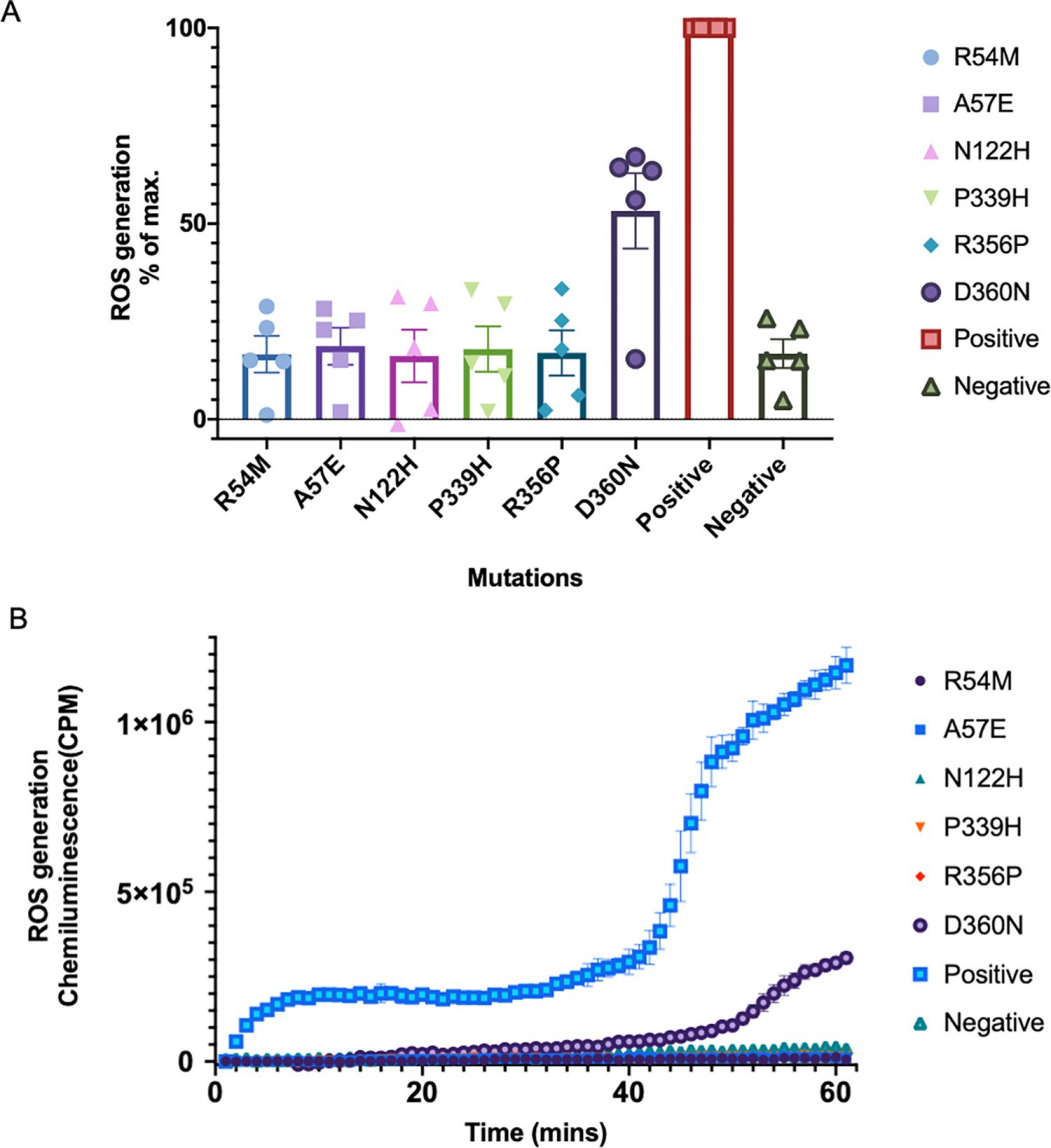
**A** ROS response in mutagenesis assays. Data are illustrated as percentages of the positive control. Positive: Wild-type NOX1; Negative: ROS generation in cells transfected with an empty plasmid. Five independent experiments were included for each mutant. **B** A representative illustration of enzyme kinetics.

We then extended the *in silico* docking results to *in vitro* mutagenesis assays. R54 and A57 are key residues of heme binding, as predicted in our docking results, and R54M and A57E were discovered in NOX2 from CGD patients. N122H was a NOX1 mutation discovered in an IBD patient. ASP122 engages in interactions with the outer-heme group. P339H and R356P were found in CGD patients with NOX2 mutations, and PRO339 and ARG356 were involved in FAD binding. D360N, a well-characterized mutation rare among healthy populations, but rather common in Ashkenazi Jewish males with ulcerative colitis (UC), D360N, also engages in active interaction with FAD in both NOX1 and NOX2 [42]. Flow cytometry analysis indicated that these mutations did not alter the level and localization of NOX1 expression on the cell surface (S3 Fig), nor the expression of NOX1 cytosolic subunits (S4 Fig). By measuring ROS generation in the mutants, we observed that most mutations of these key active residues led to a decrease in or even absence of ROS generation (Fig 5). Therefore, our *in silico* discoveries were consistent with *in vitro* experimental data and previous clinical reports, demonstrating its important clinical value.

### Mechanism of NOX1 inhibition verified with the *in silico* structure

**Fig 6:**
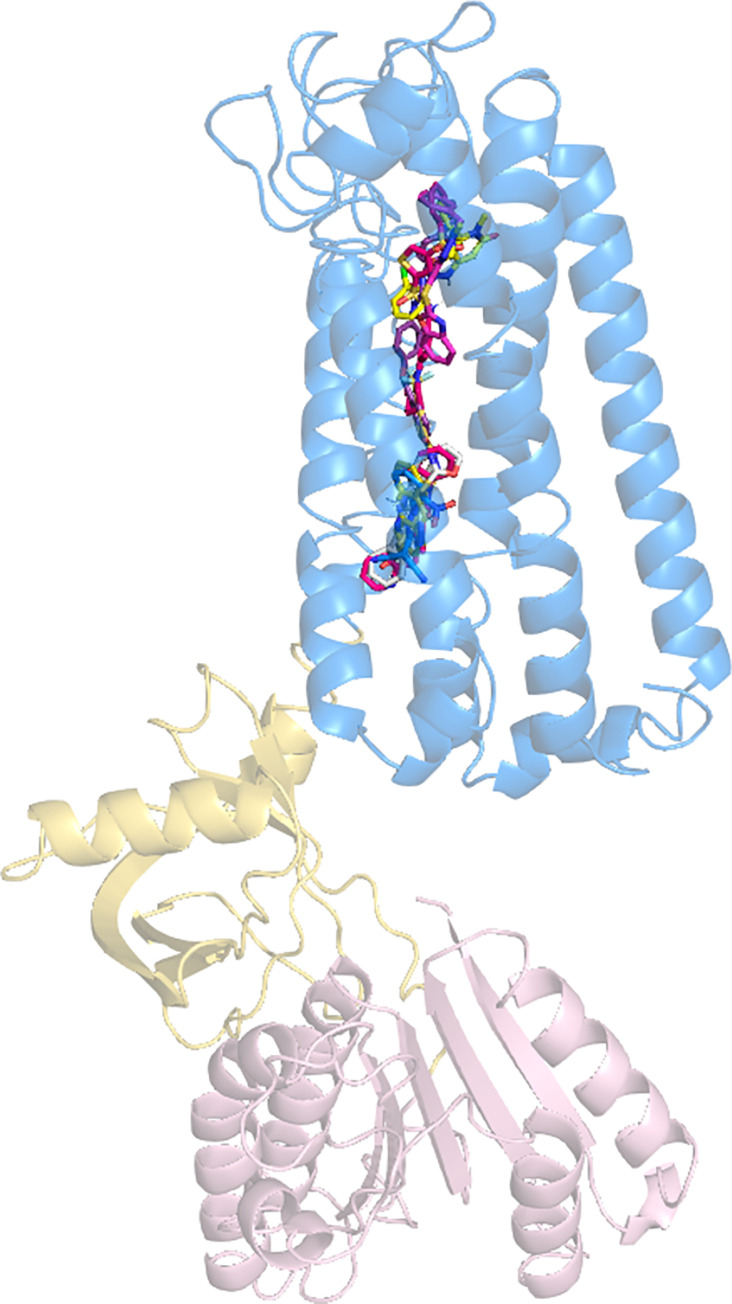
Superimposed binding scheme of the NOX1 inhibitors.

Currently, a selective NOX1/4 inhibitor (GKT137831) is undergoing a clinical trial for idiopathic pulmonary fibrosis (IPF) (ClinicalTrials.gov Identifier: NCT03865927) [43]. Several other potent inhibitors are under development for *in vivo* studies. Nevertheless, detailed mechanism of the inhibition has not been delineated. We therefore chose six reported NOX1 inhibitors as candidates for molecular docking analysis (see Table 2 for detailed information). Superimposed docking results are shown in Fig 6. It is obvious that most of the potent NOX1 inhibitors bind to the active sites for heme binding. Specifically, several amino acid residues, such as ASN122, TYR280, THR169, and ALA174 have been identified as common binding sites for NOX1 inhibitors. Therefore, to improve potency and efficacy in future drug design, these active sites identified from docking analysis would be favorable targets.

**Table 2:** Representative NOX1 inhibitors for docking analysis.

Inhibitor	Structure	Ref.
**GKT136901**		[11]
**GKT137831**		[22]
**VAS2870**		[44]
**VAS3947**		[45]
**ML090**		[46]
**ML171**		[18]

**Fig 7:**
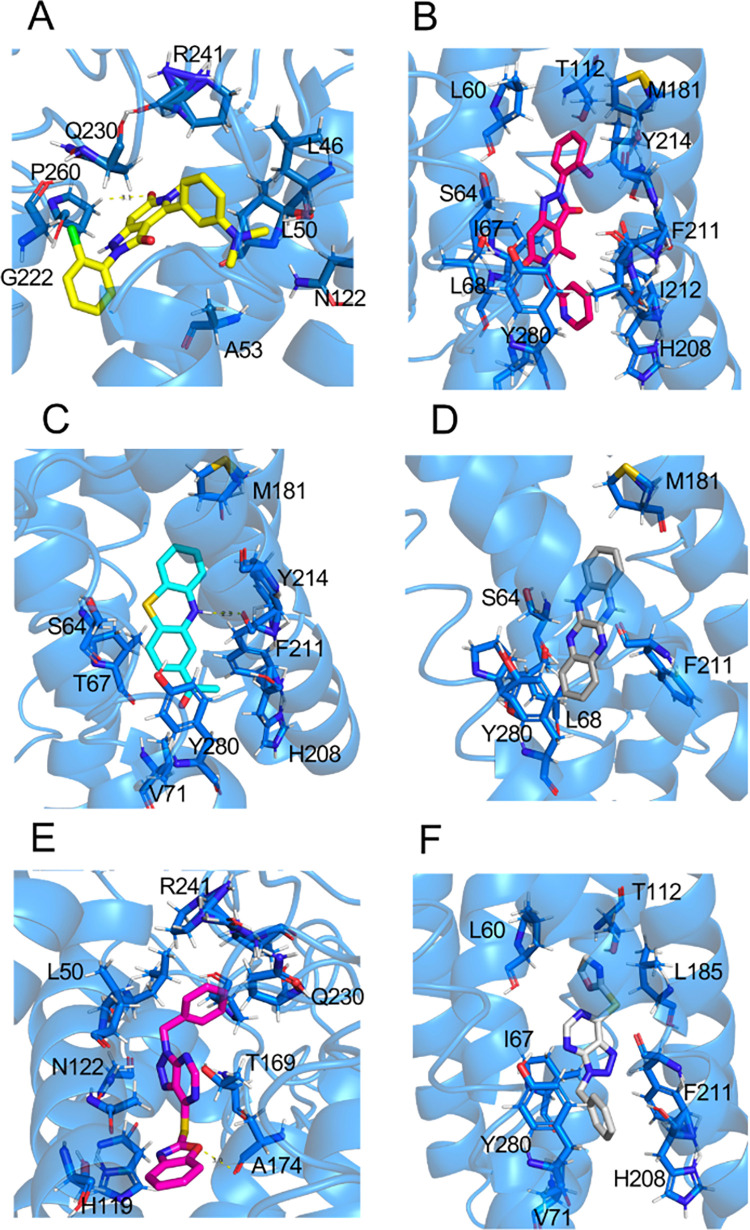
**Binding of NOX1 inhibitors deep into the center of the TM domain of NOX1, showing the binding pockets of (A)** GKT137831, **(B)** GKT136901, **(C)** ML171, **(D)** ML090, **(E)** VAS2870, **(F)** VAS3947.

GKT137831, a selective NOX1/4 inhibitor, is currently under clinical trial as a potential therapeutic of idiopathic pulmonary fibrosis (IPF). Previous studies demonstrated that GKT137831 is potent for inhibiting NOX1 with an IC_50_ of 0.14 μM [22,47,48]. Widely reported by *in vivo* studies, GKT137831 is believed to attenuate liver fibrosis, pulmonary fibrosis and hypertensive cardiac remodeling [22,48–51]. We found in our model that GKT137831 may exert its inhibitory action by filling in a pocket formed by GLY222, LEU50, ASN122, ALA53, LEU46, ARG241, THR49, PHE262, LYS261, GLY225, PRO260, GLN230 in the transmembrane domain of NOX1 (Fig 7A). In this posture, the oxygen in the purine-like structure of GKT137831 forms a hydrogen bond with PRO260, further enhancing the inhibitory effect by blocking the electron transfer. GKT136901, an analog of GKT137831, also presents its selectivity for NOX1 with an IC_50_ of 0.16 μM [11]. GKT136901, however, takes a lower position in the 6-TM region of NOX1, interacting with residues VAL71, LEU185, TYR280, TYR214, MET181, HIS208, THR112, ILE67, ILE212, LEU68, SER64, PHE211, and LEU60 (Fig 7B). Its lower inhibitory effect could result from a lack of hydrogen bonding with adjacent residues. Further pharmacological studies would be necessary to unravel the inhibitory mechanism as well as *in vivo* functions.

ML171, a novel small-molecule NOX1 inhibitor, was identified in 2010 [18,21]. The selectivity and potency of ML171 in inhibiting NOX1 was well observed (IC_50_ = 0.25 μM) [21,47]. According to our docking results, the phenothiazine-structured ML171 assumes a posture deep into the electron transfer pocket in the transmembrane region of NOX1 (Fig 7C). ML171 is surrounded by a hydrophobic pocket consisting of TYR214, TYR280, PHE211, ILE215 and LEU68. This insertion between the two heme groups is believed to impede active transport of electrons, thus preventing the generation of ROS. Notably, a nitrogen on the center ring of ML171 formed a hydrogen bond with PHE211 of NOX1 in the 6-TM region. This is believed to contribute to its low IC_50_ value of 0.25 μM. ML090, another small-molecule drug with a chemical structure similar to that of ML171, was also determined to play an active role in specific inhibition of NOX1 [46]. In our docking analysis, ML090 was found to insert into the electron transfer pocket in the transmembrane region of NOX1, similar to the mechanism of ML171 (Fig 7D). Specifically, the four-aromatic ring structure of ML090 experiences a hydrophobic interaction surrounded by TYR280, LEU68, and PHE211.

VAS2870 was discovered as a pan-NADPH oxidase inhibitor [44,52]. Preincubation with VAS2870 completely diminished the oxLDL-mediated ROS production in human endothelial cells [52]. Another study revealed that VAS2870 can attenuate chemotaxis of vascular smooth muscle cells by suppressing ROS generation [44]. Docking result supports the fact that VAS2870 can potently inhibit NOX1 by interacting with LEU50, ASN122, ARG241, TYR280 and THR169, which are key binding residues of heme group to NOX1 (Fig 7E). Moreover, a hydrogen bond is also present between a pentagon ring in VAS2870 and ALA174 residue of NOX1. Hence, we predict that VAS2870 attenuates the electron transfer process and further diminishes ROS production by NOX1. Another VAS2870 analog, VAS3947, was identified to exhibit a strong hydrophobic interaction in a pocket surrounded by PHE211, LEU185, VAL71, HIS208 and TYR280 (Fig 7F), thereby altering the normal function of heme group in active electron transport [45].

We further calculated the free energy of binding between each inhibitor and its binding sites on NOX1. For GKT137831 and GKT136901, the free energy of binding was similar (ΔG_GKT137831_ = -33.02 kcal/mol, ΔG_GKT137831_ = -37.53 kcal/mol). Likewise, another pair of structural analogs, VAS2870 and VAS3947 showed comparable free energy of binding (ΔG_VAS2870_ = -42.14 kcal/mol, ΔG_VAS3947_ = -43.63 kcal/mol). The binding was more energy-favorable for ML171 (ΔG_ML171_ = -51.44 kcal/mol) than ML090 (ΔG_ML090_ = -35.75 kcal/mol). Most of the examined inhibitors showed highly energy-favorable interactions with their binding sites. Moreover, structural analogs displayed similar binding energy when compared in pairs. Generally, the VAS- molecules were better than ML- molecules, followed by the GKT- molecules.

**Fig 8:**
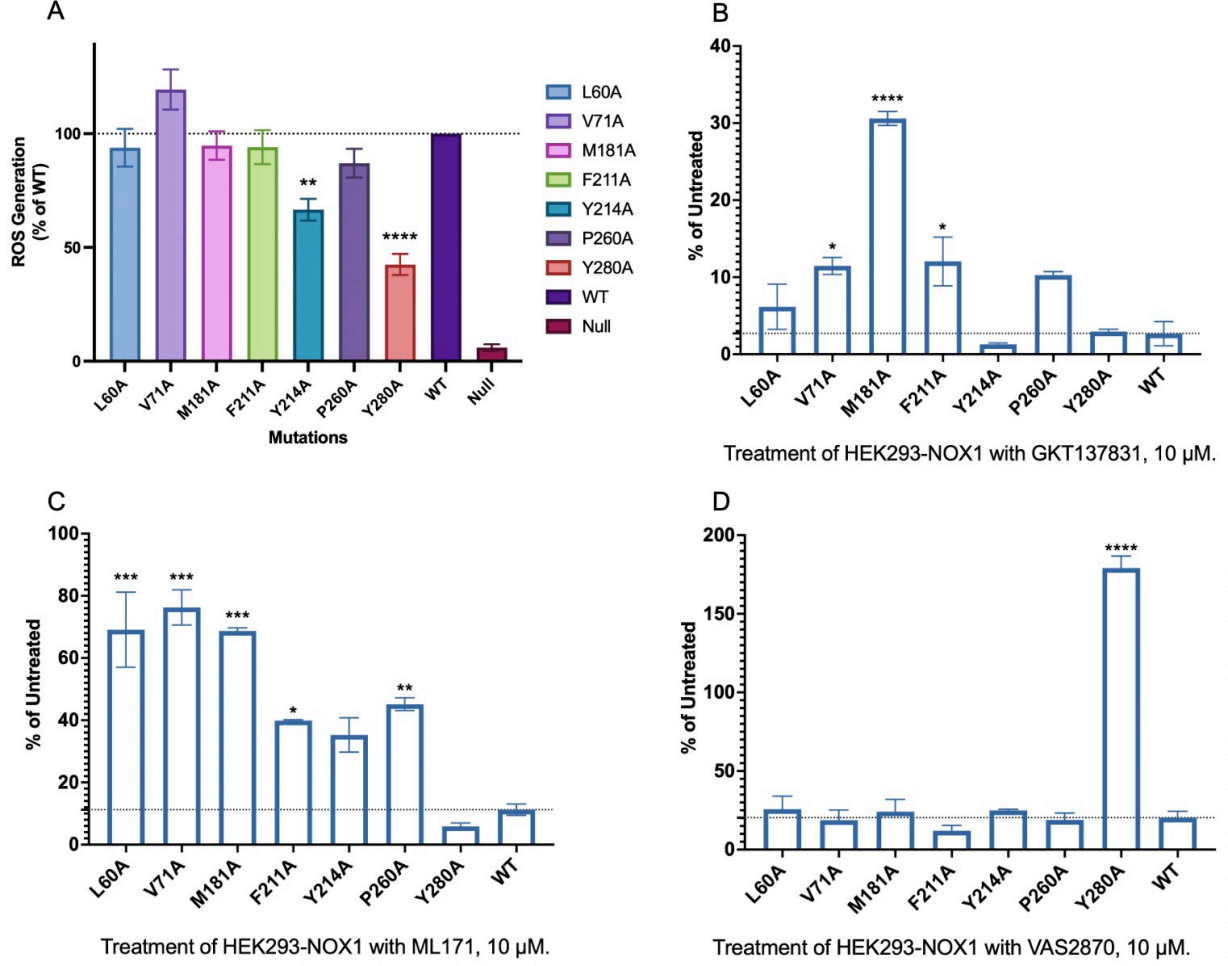
ROS inhibition assay of NOX1 mutants. **(A)** ROS generation of NOX1 wild-type and mutants without inhibitor treatment, ROS generation of WT and mutants treated with 10 μM **(B)** GKT137831, **(C)** ML171, **(D)** VAS2870.

To verify our *in silico* simulation of inhibitor binding, we next performed enzyme inhibition assays. Selected inhibitor binding sites were mutated into alanine, yet the point mutations did not cause loss of ROS generating function (Fig 8A), nor did it affect the expression of cytosolic subunits of NOX1 complex (S4 Fig). Inhibitor treatment did not cause internalization of NOX1 complex based on flow cytometric determination of cell surface expression of the NOX1 mutants (S5 Fig). We expected a significant loss of inhibition for drugs administered on those NOX1 mutants. As expected, GKT137831 treatment led to loss of inhibition of ROS on mutants V71A, M181A, F211A and P260A as compared to WT (Fig 8B). This loss of inhibitory function implies that VAL71, MET181, PHE211, PRO260 engage in GKT137831 binding to NOX1. This is consistent with our docking results, especially for the possible hydrogen bond between PRO260 and the purine oxygen in GKT137831. Similarly, the inhibitory function of ML171 was disrupted on mutants L60A, V71A, M181A, F211A, Y214A and P260A (Fig 8C). This result is in accordance with docking outcomes that ML171 is surrounded by TYR214, TYR280, PHE211, ILE215 and LEU68, implying possible interactions between PHE211 and ML171. For VAS2870, inhibition of ROS production was abrogated on Y280A mutant (Fig 8D), supporting possible hydrophobic interactions as predicted in our aforementioned molecular docking simulation results.

## Discussion

The present study deployed an *in silico* approach to build a predicted structure of NOX1. As an analog of neutrophil NADPH oxidase (NOX2), NOX1 actively participates in epithelial immunity, especially in colonic tissues. With a lack of an auto-inhibitory domain in NOXA1, NOX1 mediates constitutive release of low-level ROS, which is believed to maintain a homeostatic microenvironment in the GI tract. This corroborates with clinical observations that loss-of-function mutations of NOX1 could be attributable for the development of inflammatory bowel disease. The present study provides a structural basis for these mutational changes.

With recent development of AI, a broader application of AI in life sciences and medicine has been witnessed. In this study, we primarily utilized the RaptorX deep learning platform for protein structure prediction. For cross-validation purpose, we further compared our RaptorX-predicted NOX1 structure model with a tFold-predicted and an AlphaFold-predicted NOX1 structure models and experimentally-resolved NOX2, NOX5 and DUOX1 structures (Fig 2). Verified by molecular docking, the key binding residues of small molecule inhibitors for the predicted models are highly similar (S1 and S2 Tables). The core domains of our NOX1 model, including the 6 transmembrane region and the dehydrogenase domain, are similar to those in NOX5. In contrast, DUOX1 resembles less with our NOX1 model, probably due to lower sequence homology between the two oxidases as well as their distinct physiological functions.

Several loss-of-function mutation hotspots of NOX1 have been identified from IBD patients. As described in our study, mutations of ARG54, ASN122, and ILE167 found in IBD patients can be attributed to a disturbed conformation of the heme binding pocket. Mutations of PRO339 and ARG356, widely distributed among patients, is suggested to affect the binding of FAD to NOX1. These mutations diminish the electron transfer chain reactions, contributing to diminished ROS production. However, despite the involvement of ASP360 in FAD interactions, we did not observe a complete depletion of ROS generation with the mutant D360N, nor widely-distributed disease phenotype in population with this mutation. This could be explained by a strong interaction led by hydrogen bonds formed extensively between the substrate and the other residues in the binding pocket. Similarly, only frameshift mutation at site D360, but rarely substitutions, has been characterized in X-CGD patients. Mutation at this very site of NOX2 may not be adequate to eliminate ROS production and cause CGD symptoms. Therefore, with such a strong interaction inside the FAD active site, mutations resulted in a decrease of ROS generation to a much less extent than mutations of amino acids in the heme binding pockets.

On the contrary, an excessive expression of NOX1 may correlate with cancers of multiple epithelial tissues, acute lung inflammation and tissue fibrogenesis. Over-expressed NOX1 may lead to an excessive amount of ROS generated in the local tissues. Accumulation of free radicals may lead to tissue damage, carcinogenesis and fibrosis. Recent *in vivo* and *in vitro* evidence indicates that NOX1-derived ROS may underlie enhanced vasoconstrictions in response to angiotensin II in arterial hypertension and vascular diseases [53,54]. In contrast, several *in vivo* reports pointed out that knocking down of NOX1 did not affect the blood pressure and the development of hypertension [9,55,56]. Despite lacking of such clinical evidence in patients, selective downregulation of NOX1 also have a role in treatment of reperfusion injury in patients with atherosclerosis [57]. Similar to complications in vascular smooth muscle cells, NOX1 also plays a role in tissue fibrosis, including liver fibrosis and pulmonary fibrosis [22,58,59]. Indeed, the small molecule inhibitor covered in this study, GKT137831, is also under a clinical trial of idiopathic pulmonary fibrosis [59]. New findings revealed that NOX1 inhibition may be applicable for neurodegenerative disorders causing dementia. In a recent report, Nortley *et al* discovered that in both experimental and clinical manifestation of Alzheimer’s disease, β-amyloid may induce ROS in a NOX1/4-dependent fashion, leading to cerebral hypertension [60].

To address these health problems, potent NOX1 inhibitors have been developed. Our molecular docking analysis on the predicted structure and *in vitro* ROS inhibition assays shed light on the potential mechanisms for these potent NOX1 inhibitors. Most NOX1 inhibitors, including GKT137831 which is under clinical trials, show strong interaction within the 6-TM region of NOX1, diminishing electron transport between the two heme groups and towards the extracellular ROS generation. In particular, through *in silico* simulation and *in vitro* functional verifications, LEU60, VAL71, MET181, LEU185, HIS208, PHE211, TYR214, and TYR280 are identified as the main inhibitory active sites for a potent and selective inhibition of NOX1. The predicted roles of these amino acids in the binding of the three NOX1 inhibitors were verified through our site-directed mutagenesis studies, which showed reduced inhibition when these amino acids were replaced with alanine. As NOXA1 and NOXO1 are both key players in NOX1 complex formation and ROS generation, we further examined the expression levels of NOXA1 and NOXO1 and excluded the possibility that the inhibitory functions of different inhibitors could be masked by different expression levels of cytosolic subunits of the NOX1 complex [3,61,62]. As for future drug development, structure-based drug designs may take advantage of the proposed inhibition mechanism for better identification and development of novel specific therapeutics against diseases related to overexpression of NOX1.

Under the current COVID-19 pandemic, therapeutics inhibiting NOX1 activity may be protective against acute lung injury and acute respiratory distress syndrome, which are common complications in severe patients of COVID-19. Evidence has been accumulating that COVID-19 is a multi-systemic inflammation posing greatest threats to epithelial and endothelial tissues, where NOX1 is enriched [63–66]. NOX1 may hence be a potential drug target against COVID-19 for its severe symptoms. Nevertheless, some reports illustrated a totally different role of NOX1 in pulmonary infections. A previous study used an influenza A virus challenge model to identify that NOX1 suppresses influenza A virus-induced lung inflammation, yet the isoform sibling NOX2 presents a pro-inflammatory role in influenza A virus infection [67]. Understanding the structural basis for NOX inhibition may help solving puzzles of this type.

Owing to difficulties in protein expression, purification and further crystallization or cryo-EM-based structural analysis, efforts have been made to use computational tools to facilitate protein structure prediction and consequent drug development. Previous advances in homology modeling have predicted a large number of protein structures, some of which were further resolved with crystallography or cryo-EM-based analysis and those predicted structures were proven to be effectively correct. Striking development in artificial intelligence and deep learning has made it possible for profound and accurate protein structure prediction now. In this study, we utilized well-developed artificial intelligence tools based on deep learning algorithms to predict the 3D structure of NOX1. Serendipitously, this RaptorX-predicted structure, verified in parallel with two other deep learning-predicted models, fits known knowledge of active NOX1 domains engaged in electron transfer and ROS generation. The predicted structure was further validated through a series of molecular docking and *in vitro* experiment procedures. We therefore believe that the structure predicted is of high confidence and accuracy. Next, by performing molecular docking with several reported NOX1 inhibitors, we tried to explain the inhibitory mechanisms of NOX1 with respect to our predicted structure. Our study provides a possibility to utilize *in silico* structure predicted by artificial intelligence for further structural studies and drug development. Moreover, an open-source AI protein structure prediction tool, AlphaFold 2, brings improved accuracy that will benefit structure-based drug screening and novel design of therapeutics, especially for diseases related to proteins without a resolved structure [31,68]. It is expected that NOX1-based therapeutics will eventually benefit patients with epithelial immunity impairments.

## Supporting information

S1 Fig Local contact map of the predicted NOX1 structure model. Protein contacts were predicted and visualized using RaptorX.
(PDF)

S2 Fig Electron transport channels in predicted NOX1 6-TM domain. Channels were visualized with default settings using CAVER PyMOL 3.0 Plugin.
(PDF)

S3 Fig Cell surface expression of NOX1 mutants. Wild-type NOX1 and its mutants were transiently expressed in HEK293 cells. After 24 h, cell surface expressed NOX1 was labelled by an anti-NOX1 FITC antibody and analyzed by flow cytometry. Mean fluorescence intensity was collected and analyzed for NOX1 expression. Baseline signals were detected in antibody-stained cells transfected with empty plasmids (negative control). Relative expression level was calculated as the percentage ratio against the wild-type NOX1 (100%, indicated by the dotted line). Data shown are means ± SEM of three independent experiments.
(PDF)

S4 Fig Expression of NOXA1 and NOXO1 in cells transfected with wild-type NOX1/mutants. Wild-type NOX1 and its mutants, along with NOXA1 and NOXO1, were transiently expressed in HEK293 cells. Western blotting has been applied to detect the expression levels of NOXA1 and NOXO1 in cells expressing wild-type NOX1 and mutants. β-actin levels were detected for reference. (A) Representative blotting images were chosen among results from three independent experiments. (B) Quantification of the immunoblot protein bands. Densities were calculated for protein expression levels from three independent western blotting experiments by Image J software. Two-way ANOVA was performed and all comparisons showed no statistical significance.
(PDF)

S5 Fig Cell surface expression of NOX1 upon inhibitor treatments. Wild-type NOX1 and its mutants, along with NOXA1 and NOXO1, were transiently expressed in HEK293 cells. Samples were treated with 10 μM small-molecule inhibitors or HBSS control, respectively for 30 mins prior to antibody staining. Cell surface expressed NOX1 was labeled by an anti-NOX1 FITC antibody and analyzed by flow cytometry. Mean fluorescence intensity was analyzed for NOX1 expression. Relative expression level was calculated as the percentage ratio against the sample of wild-type/mutant NOX1 treated with HBSS (100%, indicated by the dotted line). Data shown are means ± SEM of three independent experiments. Two way ANOVA was performed and comparison test results are attached in the table. ns stands for not significant, whereas the *p* values are listed for significant comparisons.
(PDF)

S1 Table Summary of binding sites of tFold-predicted NOX1 structure model. (PDF)

S2 Table Summary of small-molecule inhibitor binding sites of AlphaFold-predicted NOX1 structure model. (PDF)
