# Supplementary material for: A predicted structure of NADPH Oxidase 1 identifies key components of ROS generation and strategies for inhibition
Source: PLoS One. 2023 May 3;18(5):e0285206. doi: 10.1371/journal.pone.0285206 (PMC10155968; doi:10.1371/journal.pone.0285206)
Supplement: S1 Fig — Protein contacts were predicted and visualized using RaptorX. (PDF) [file pone.0285206.s001.pdf]

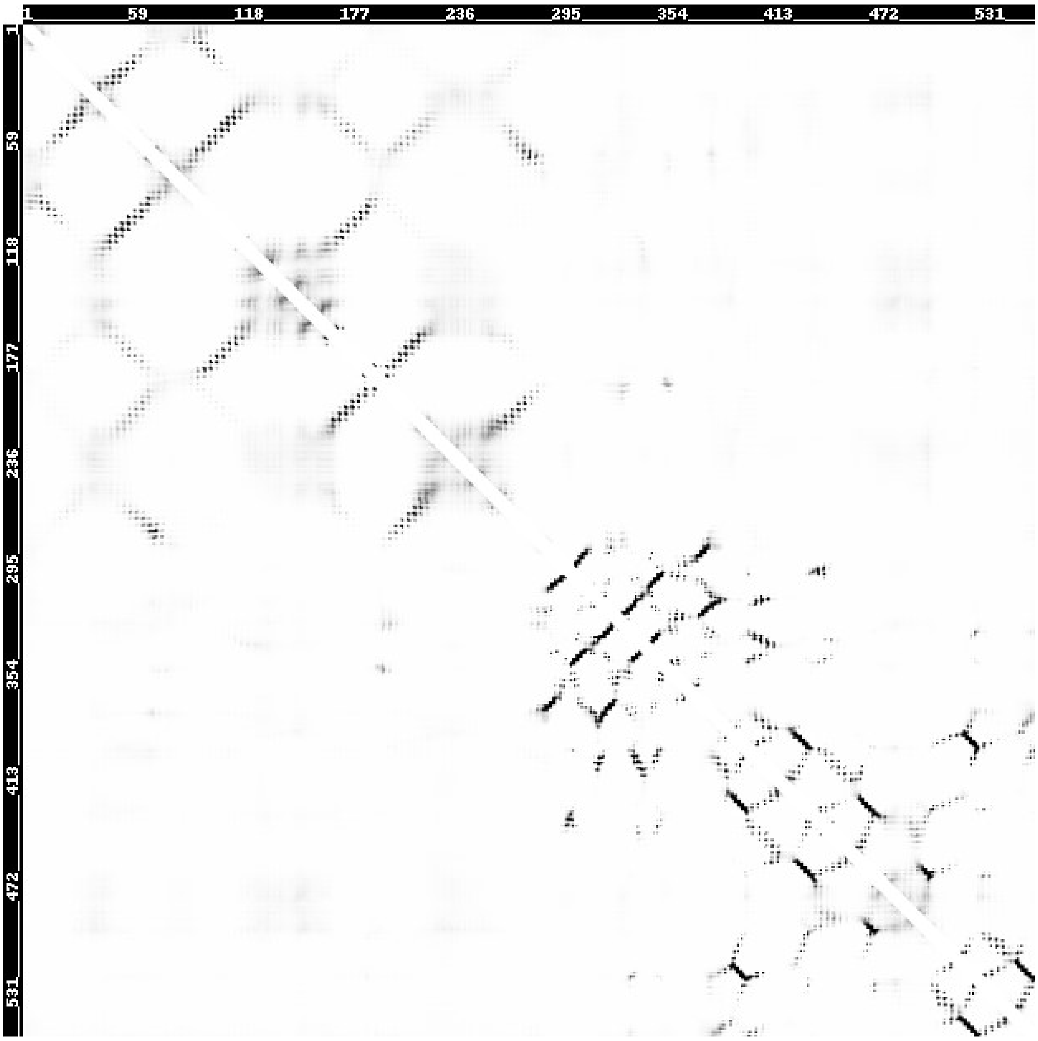

S1 Fig. Local contact map of the predicted NOX1 structure model. Protein contacts were predicted and visualized using RaptorX.
