# Supplementary material for: A predicted structure of NADPH Oxidase 1 identifies key components of ROS generation and strategies for inhibition
Source: PLoS One. 2023 May 3;18(5):e0285206. doi: 10.1371/journal.pone.0285206 (PMC10155968; doi:10.1371/journal.pone.0285206)
Supplement: S2 Fig — Channels were visualized with default settings using CAVER PyMOL 3.0 Plugin. (PDF) [file pone.0285206.s002.pdf]

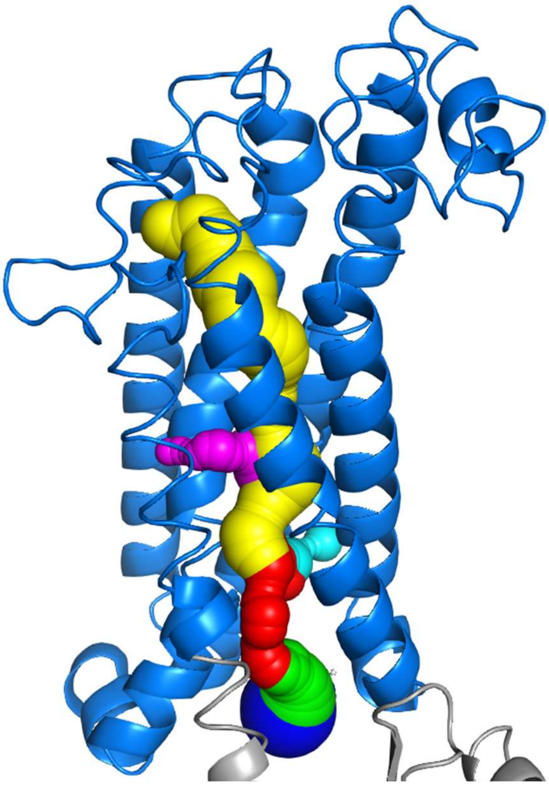

S2 Fig. Electron transport channels in predicted NOX1 6-TM domain. Channels were visualized with default settings using CAVER PyMOL 3.0 Plugin.
