# Supplementary material for: A predicted structure of NADPH Oxidase 1 identifies key components of ROS generation and strategies for inhibition
Source: PLoS One. 2023 May 3;18(5):e0285206. doi: 10.1371/journal.pone.0285206 (PMC10155968; doi:10.1371/journal.pone.0285206)
Supplement: S3 Fig — Wild-type NOX1 and its mutants were transiently expressed in HEK293 cells. After 24 h, cell surface expressed NOX1 was labelled by an anti-NOX1 FITC antibody and analyzed by flow cytometry. Mean fluorescence intensity was collected and analyzed for NOX1 expression. Baseline signals were detected in antibody-stained cells transfected with empty plasmids (negative control). Relative expression level was calculated as the percentage ratio against the wild-type NOX1 (100%, indicated by the dotted line). Data shown are means ± SEM of three independent experiments. (PDF) [file pone.0285206.s003.pdf]

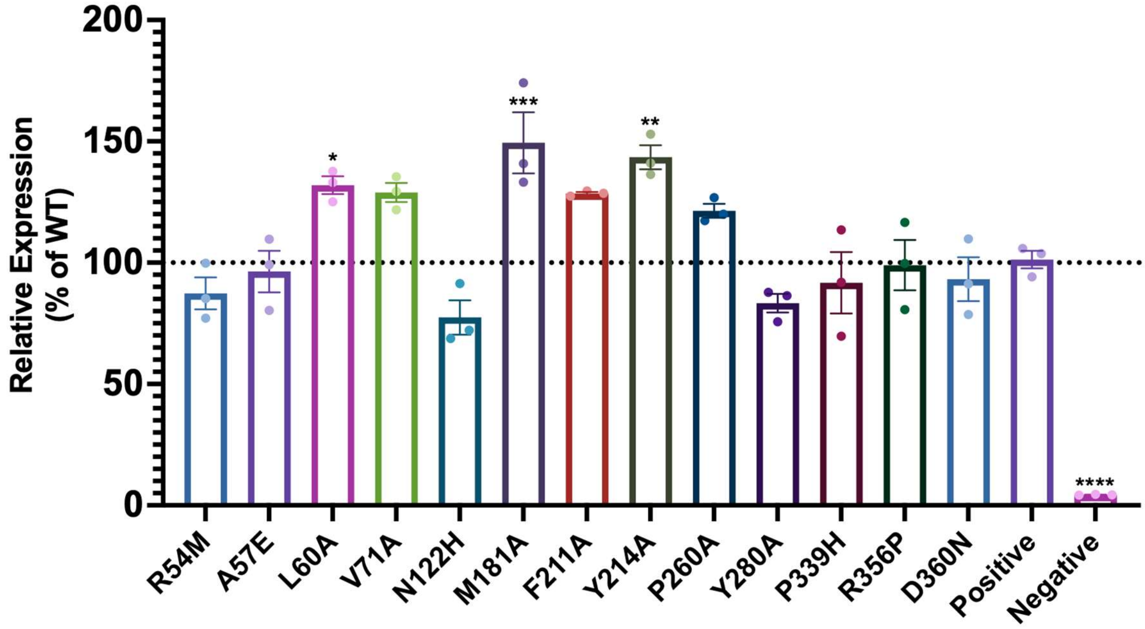

S3 Fig. Cell surface expression of NOX1 mutants. Wild-type NOX1 and its mutants were transiently expressed in HEK293 cells. After 24 h, cell surface expressed NOX1 was labelled by an anti-NOX1 FITC antibody and analyzed by flow cytometry. Mean fluorescence intensity was collected and analyzed for NOX1 expression. Baseline signals were detected in antibody-stained cells transfected with empty plasmids (negative control). Relative expression level was calculated as the percentage ratio against the wild-type NOX1 (100%, indicated by the dotted line). Data shown are means ± SEM of three independent experiments.
