# Supplementary material for: A predicted structure of NADPH Oxidase 1 identifies key components of ROS generation and strategies for inhibition
Source: PLoS One. 2023 May 3;18(5):e0285206. doi: 10.1371/journal.pone.0285206 (PMC10155968; doi:10.1371/journal.pone.0285206)
Supplement: S4 Fig — Wild-type NOX1 and its mutants, along with NOXA1 and NOXO1, were transiently expressed in HEK293 cells. Western blotting has been applied to detect the expression levels of NOXA1 and NOXO1 in cells expressing wild-type NOX1 and mutants. β-actin levels were detected for reference. (A) Representative blotting images were chosen among results from three independent experiments. (B) Quantification of the immunoblot protein bands. Densities were calculated for protein expression levels from three independent western blotting experiments by Image J software. Two-way ANOVA was performed and all comparisons showed no statistical significance. (PDF) [file pone.0285206.s004.pdf]

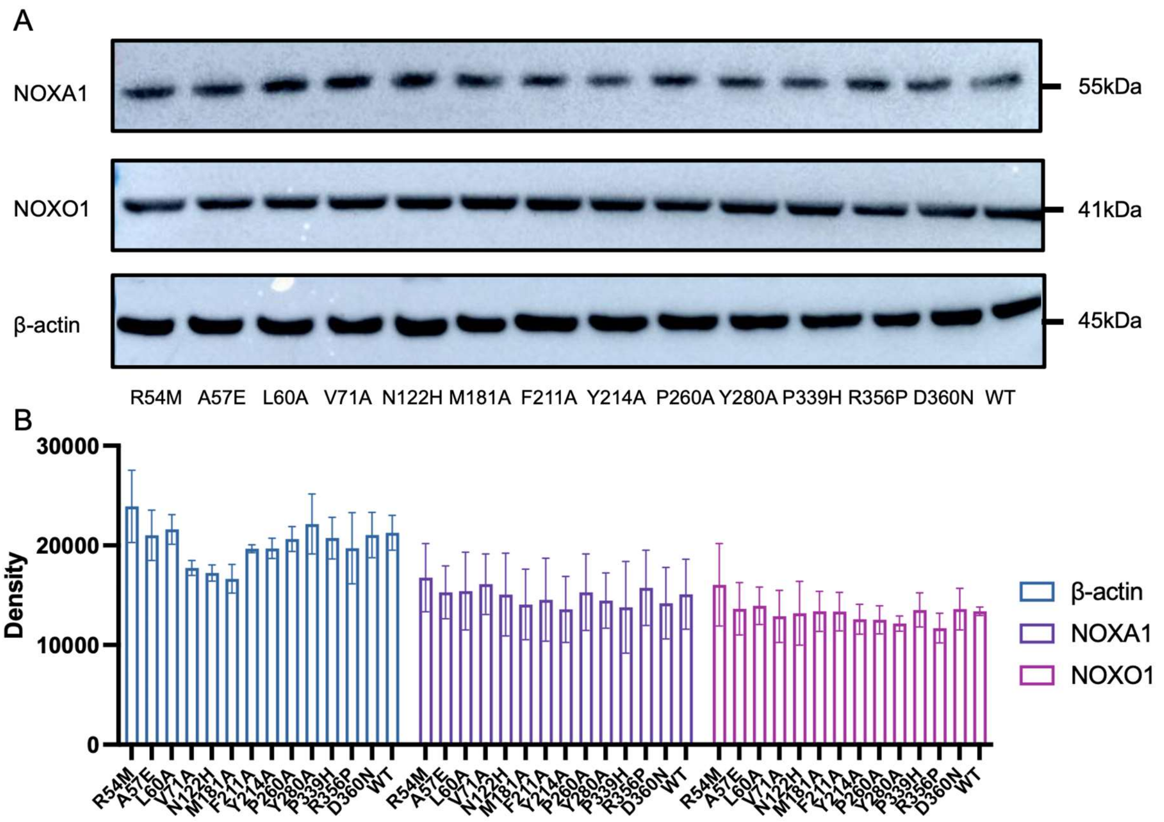

S4 Fig. Expression of NOXA1 and NOXO1 in cells transfected with wild-type NOX1/mutants. Wild-type NOX1 and its mutants, along with NOXA1 and NOXO1, were transiently expressed in HEK293 cells. Western blotting has been applied to detect the expression levels of NOXA1 and NOXO1 in cells expressing wild-type NOX1 and mutants. β-actin levels were detected for reference. **(A)** Representative blotting images were chosen among results from three independent experiments. **(B)** Quantification of the immunoblot protein bands. Densities were calculated for protein expression levels from three independent western blotting experiments by Image J software. Two-way ANOVA was performed and all comparisons showed no statistical significance.
