# Supplementary material for: A predicted structure of NADPH Oxidase 1 identifies key components of ROS generation and strategies for inhibition
Source: PLoS One. 2023 May 3;18(5):e0285206. doi: 10.1371/journal.pone.0285206 (PMC10155968; doi:10.1371/journal.pone.0285206)
Supplement: S5 Fig — Wild-type NOX1 and its mutants, along with NOXA1 and NOXO1, were transiently expressed in HEK293 cells. Samples were treated with 10 μM small-molecule inhibitors or HBSS control, respectively for 30 mins prior to antibody staining. Cell surface expressed NOX1 was labeled by an anti-NOX1 FITC antibody and analyzed by flow cytometry. Mean fluorescence intensity was analyzed for NOX1 expression. Relative expression level was calculated as the percentage ratio against the sample of wild-type/mutant NOX1 treated with HBSS (100%, indicated by the dotted line). Data shown are means ± SEM of three independent experiments. Two way ANOVA was performed and comparison test results are attached in the table. ns stands for not significant, whereas the p values are listed for significant comparisons. (PDF) [file pone.0285206.s005.pdf]

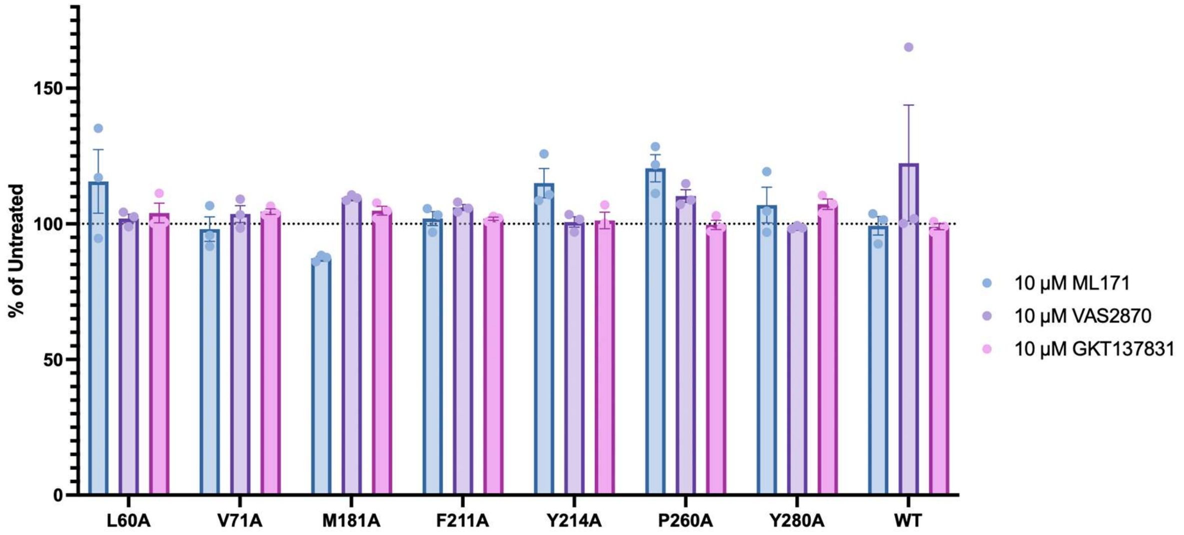

	Treated_10 μM ML171	Treated_10 μM VAS2870	Treated_10 μM GKT137831
WT vs. L60A	ns	* (p=0.0279)	ns
WT vs. V71A	ns	ns	ns
WT vs. M181A	ns	ns	ns
WT vs. F211A	ns	ns	ns
WT vs. Y214A	ns	* (p=0.0172)	ns
WT vs. P260A	* (p=0.0208)	ns	ns
WT vs. Y280A	ns	** (p=0.0076)	ns

S5 Fig. Cell surface expression of NOX1 upon inhibitor treatments. Wild-type NOX1 and its mutants, along with NOXA1 and NOXO1, were transiently expressed in HEK293 cells. Samples were treated with 10 μM small-molecule inhibitors or HBSS control, respectively for 30 mins prior to antibody staining. Cell surface expressed NOX1 was labeled by an anti-NOX1 FITC antibody and analyzed by flow cytometry. Mean fluorescence intensity was analyzed for NOX1 expression. Relative expression level was calculated as the percentage ratio against the sample of wild-type/mutant NOX1 treated with HBSS (100%, indicated by the dotted line). Data shown are means ± SEM of three independent experiments. Two-way ANOVA was performed and comparison test results are attached in the table. ns stands for not significant, whereas the *p* values are listed for significant comparisons.
