# Supplementary material for: A predicted structure of NADPH Oxidase 1 identifies key components of ROS generation and strategies for inhibition
Source: PLoS One. 2023 May 3;18(5):e0285206. doi: 10.1371/journal.pone.0285206 (PMC10155968; doi:10.1371/journal.pone.0285206)
Supplement: S1 Table — (PDF) [file pone.0285206.s006.pdf]

S1 Table. Summary of binding sites of tFold-predicted NOX1 structure model.

Molecules	Binding Sites
Heme1	ARG54, ARG57, ASN122, ARG258
Heme2	HIS101, HIS208, ARG197, PHE204, LYS98, LYS102
FAD	ARG356, PRO533, PRO339, ILE405
NADPH	ASN450, SER454, ALA345, GLU348, LEU491
GKT136901	LEU68, VAL71, ARG73, PHE211, TRP205, TYR280, ARG284
GKT137831	LEU50, GLN230, ASN122, PRO260, GLU263, PHE262
VAS2870	VAL71, ARG73, TRP205, PHE211, TYR280, ARG284, ARG287
VAS3947	LEU50, ARG54, PRO260, PHE262, LEU58, GLN230
ML090	ARG54, PHE211, TYR214, PHE262, TYR280
ML171	ILE67, SER64, ALA192, HIS208, PHE211, TYR214
