# Supplementary material for: A predicted structure of NADPH Oxidase 1 identifies key components of ROS generation and strategies for inhibition
Source: PLoS One. 2023 May 3;18(5):e0285206. doi: 10.1371/journal.pone.0285206 (PMC10155968; doi:10.1371/journal.pone.0285206)
Supplement: S2 Table — (PDF) [file pone.0285206.s007.pdf]

**S2 Table. Summary of small-molecule inhibitor binding sites of AlphaFold-predicted NOX1 structure model.**

| **Molecules** | **Binding Sites** |
|---|---|
| Heme1 | ARG54, ARG57, PRO260, ALA174, THR169, ASN122 |
| Heme2 | HIS208, HIS101, PHE204, ARG73, TRP337 |
| FAD | TRP337, PRO339, ARG356, PHE564, ILE405, PRO533 |
| NADPH | LEU451, TRP418, ASN450, ALA345 |
| GKT136901 | LEU68, ARG73, TRP205, HIS208, PHE211, TYR280, ARG284 |
| GKT137831 | VAL71, ARG73, TYR280, ARG284, ARG287 |
| VAS2870 | VAL71, ARG73, TRP205, PHE211, TYR280, ARG284, ARG287 |
| VAS3947 | VAL71, ARG73, TRP205, TYR280, ARG284, ARG287, TRP337 |
| ML090 | LEU60, HIS115, GLY178, MET181, THR182, TYR214, HIS221 |
| ML171 | ILE67, HIS101, LEU185, HIS208, PHE211, TYR214, TYR280 |
